# X-ray data about the structural response of melt-spun poly(3-hydroxybutyrate) fibers to stress and temperature

**DOI:** 10.1016/j.dib.2020.105675

**Published:** 2020-05-11

**Authors:** Edith Perret, Felix A. Reifler, Ali Gooneie, Kang Chen, Figen Selli, Rudolf Hufenus

**Affiliations:** a Laboratory for Advanced Fibers, Empa, Swiss Federal Laboratories for Materials Science and Technology, Lerchenfeldstrasse 5, 9014 St. Gallen, Switzerland; b Center for X-ray Analytics, Empa, Swiss Federal Laboratories for Materials Science and Technology, Überlandstrasse 129, 8600 Dübendorf, Switzerland; c State Key Laboratory for Modification of Chemical Fibers and Polymer Materials, College of Materials Science and Engineering, Donghua University, Shanghai 201620, China; d Dokuz Eylul University, Department of Textile Engineering, Izmir, Turkey

**Keywords:** Wide-angle x-ray diffraction, small-angle x-ray scattering, P3HB, poly(3-hydroxybutyrate), biodegradable, melt-spun, stress annealing, fiber

## Abstract

Mechanical properties of as-spun, aged and stress-annealed melt-spun poly(3-hydroxybutyrate) (P3HB) fibers are presented in section 1.1. Section 1.2 presents tables with stress/temperature conditions and exposure times during in-situ laboratory WAXD and SAXS experiments, and section 1.3 presents azimuthal profiles of the corresponding WAXD patterns with extracted orientation factors of the α-crystals. Section 1.4 presents the extracted long-spacings, coherence lengths and crystal sizes from SAXS patterns. The corresponding fits of meridional and transversal SAXS profiles are shown in sections 1.5 and 1.6, respectively. In-situ synchrotron measurements during tensile drawing of differently pre-annealed P3HB fibers are presented in section 1.7. A detailed description of the tensile, SAXS/WAXD measurements and analysis is given in the experimental section 2. The laboratory SAXS/WAXD measurements during stress annealing were performed with a Bruker Nanostar U diffractometer (Bruker AXS, Karlsruhe, Germany) and a heating stage H+300 (Bruker AXS, Germany). Different weights were attached to the fibers during heating to apply stress. The synchrotron measurements during tensile drawing were performed at the cSAXS beamline at the Swiss Light Source of the Paul Scherrer Institute in Switzerland. The fibers were drawn with a TS 600 tensile stage (Anton Paar GmbH, Austria) using a 5 N load cell. For more information see 'Structural response of melt-spun poly(3-hydroxybutyrate) fibers to stress and temperature' [1].

Specifications Table

**Table:** 

Subject	Materials Science: Polymers and Plastics
Specific subject area	Biodegradable melt-spun monofilaments.
Type of data	Table Image Figure Equations
How data were acquired	Instruments: Bruker Nanostar U diffractometer Synchrotron X-ray measurements (cSAXS beamline, PSI, Switzerland) Software: DIFFRAC.EVA (version 4.2., Bruker AXS, Karlsruhe, Germany) Python codes
Data format	Raw Analyzed
Parameters for data collection	Wide-angle x-ray diffraction (WAXD) and small-angle x-ray scattering (SAXS) patterns were taken in-situ during stress annealing of melt-spun P3HB fibers. Synchrotron WAXD and SAXS patterns were measured during tensile drawing of pre-annealed P3HB fibers.
Description of data collection	WAXD and SAXS patterns during in-situ stress annealing of three to four years aged fibers were recorded on a Bruker Nanostar U diffractometer (Bruker AXS, Germany) with Cu K_α_ radiation (λ = 1.5419 Å) and a VÅNTEC-2000 MikroGap area detection system. A beam defining pinhole of 300 µm was used. The WAXD and SAXS measurements were performed in two separate experiments with distances of 17.1 cm and 96.3 cm, respectively, between sample and active detector area. The heating stage H+300 (Bruker AXS, Germany) of the Nanostar diffractometer was used in order to study the effect of annealing. Single filaments were mounted on a custom-made fiber holder and different weights were attached at the end of the filaments in order to study the combined influence of heat and tension. In-situ WAXD and SAXS measurements during tensile drawing of pre-annealed P3HB fibers were performed at the cSAXS beamline at the Swiss Light Source of the Paul Scherrer Institute in Switzerland.
Data source location	Empa, St. Gallen, Switzerland
Data accessibility	Mendeley Data DOI: 10.17632/38twf5trnw.1
Related research article	Edith Perret, Felix A. Reifler, Ali Gooneie, Kang Chen, Figen Selli, Rudolf Hufenus Structural response of melt-spun poly(3-hydroxybutyrate) fibers to stress and temperature Polymer DOI: 10.1016/j.polymer.2020.122503

## Value of the data

• The understanding of the structure-property relationship in P3HB fibers is of great value for biomedical applications.
• The detailed description of WAXD and anisotropic 2D SAXS pattern data analysis of melt-spun fibers is potentially useful to other researchers.
• The detailed description of in-situ synchrotron measurements during tensile drawing of P3HB fibers is potentially useful to other researchers.
• The presented x-ray data is of great interest to the field of bio-polymers and is useful for the further development of melt-spinning of P3HB fibers.
• The reported structural parameters and mechanical properties of stress-annealed P3HB fibers may serve as a reference for other authors.

## 1 Data Description

### 1.1 Mechanical properties of as-spun, aged and stress-annealed fibers

The mechanical properties of as-spun and aged fibers are given in Table 1.

**Table 1:** Mechanical properties of as-spun and aged fiber.

Aging time	Ultimate tensile stress (MPa)	Elongation at break (%)	Toughness (MPa)
as-spun	154±9	25±1	24±2
7 years	148±14	25±2	25±2

The mechanical properties of stress-annealed fibers are given in Table 2.

**Table 2:** Mechanical properties of stress-annealed fibers. Colors correspond to the color scheme used in Fig. 1 of the publication by E. Perret et al. [1].

**Fig. 1:**
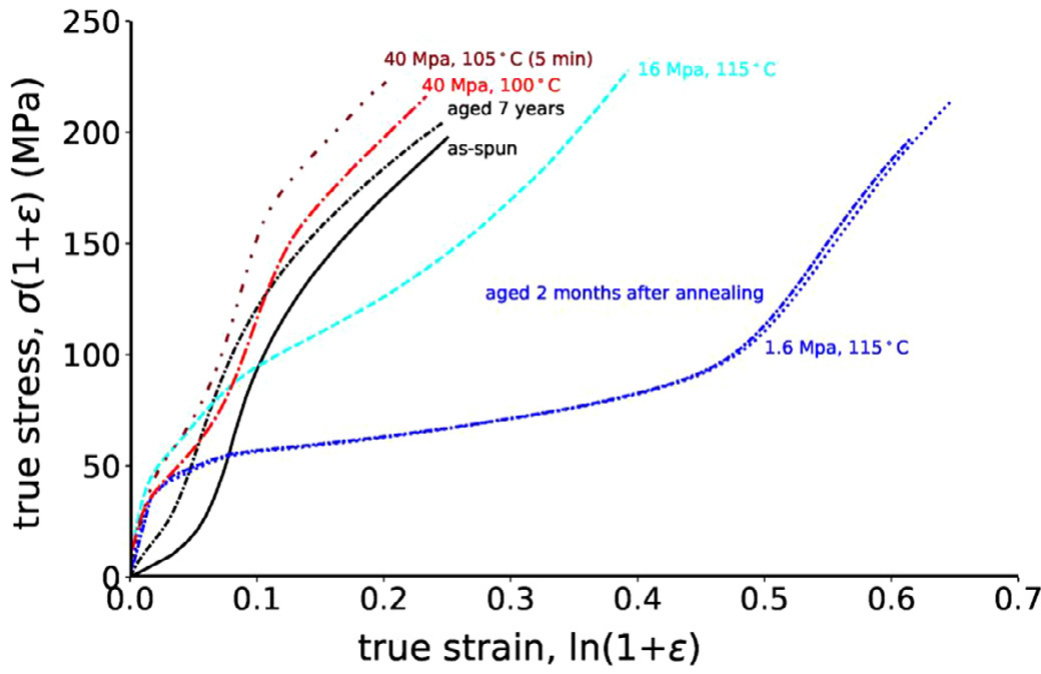
True stress-strain curves of as-spun, aged and stress-annealed P3HB fibers.

**Fig. 2:**
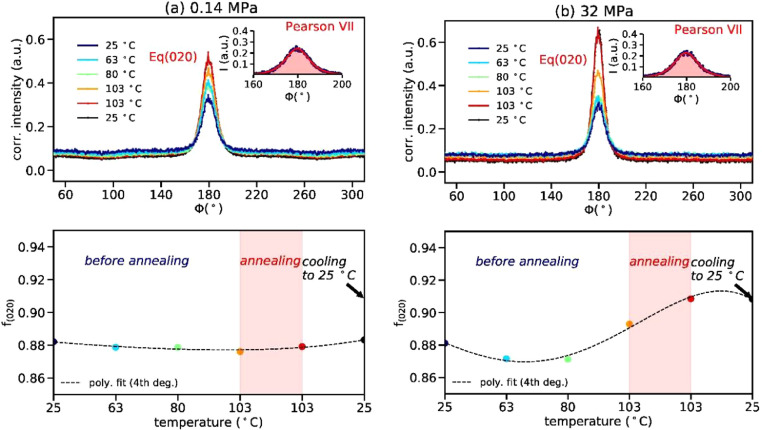
Upper row: (a) Azimuthal profiles of the (020) annulus for the filaments with applied tensile stress 0.14 MPa (a) and 32 MPa (b) for different temperatures. The Φ angles of 180° and 270° correspond to the equatorial and the meridional direction, respectively. The insets show fitted Pearson VII curves to the Eq(020) peaks. The background in the azimuthal profiles arises from randomly oriented crystals and the amorphous phase. Lower row: Corresponding orientation factors of the highly oriented (020) planes as a function of the temperature sequence.

True stress-strain curves that have been converted from Fig. 2a of the publication by E. Perret et al. [1] using the formulas given in section 2.1 are shown in Fig. 1. The ultimate true stress values reflect the maximum internal load that can be tolerated by the stretched chains within the fibers. Thus, these values are all quite similar for all pre-annealed fibers.

### 1.2 In-situ laboratory WAXD and SAXS during stress-annealing: tables with experimental conditions

Stress/temperature conditions and exposure times during in-situ laboratory WAXD and SAXS measurements are given in Table 3 and Table 4.

**Table 3:** Conditions during in-situ WAXD measurements for all WAXD patterns that were used by E. Perret et al. [1].

Stress (MPa)	Annealing temp. (°C)	Exposure time (s)	Scan number	Time stamp (h:min)
0	25	1800	#296	0
0	45	1800	#298	0:37
0	63	1800	#302	2:10
0	80	1800	#305	3:11
0	103	3600	#308	4:41
0	103	3600	#315	11:43
0	25	7200	#326	22:50
32	25	1800	#331	0
32	46	1800	#334	1:00
32	63	1800	#337	1:54
32	80	1800	#340	2:54
32	103	3600	#343	4:25
32	103	3600	#350	11:27
32	25	1800	#359	13:58

**Table 4:** Conditions during in-situ SAXS measurements for all SAXS patterns that were used by E. Perret et al. [1].

Stress (MPa)	Annealing temp. (°C)	Exposure time (s)	Scan number	Time stamp (h:min)
0	25	7200	#217	0
0	45	1800	#218	0:52
0	61	1800	#219	1:43
0	90	1800	#221	2:53
0	115	7200	#224	5:59
0	70	1800	#226	7:14
0	25	7200	#231	13:54
32	25	7200	#201	0
32	45	1800	#202	0:55
32	61	1800	#203	1:43
32	90	1800	#205	2:51
32	115	7200	#208	5:58
32	70	1800	#210	7:13
32	25	7200	#215	13:53

### 1.3 In-situ laboratory WAXD: azimuthal profiles

We have extracted the azimuthal profiles of the (020) annulus of the WAXD pattern taken from the filaments during low-stress annealing (Fig. 2a) and during high-stress annealing (Fig. 2b). The orientation factors of the highly oriented (020) planes are shown in the lower row of Fig. 2. The background, which arises from randomly oriented crystals and the amorphous phase, decreases with increasing temperature, and the equatorial intensity (020) increases and remains after the cool-down to 25°C. The increase in the intensity of the equatorial reflection from (020) during the annealing is a result of a redistribution of the intensity from the background into the peak.

The orientation factor of the low-stress annealed filament is not significantly changing, as is seen in the lower row of Fig. 2a. An increase in the orientation of the crystals, however, is observed above 80 °C for the high-stress annealed filament (Fig. 2b).

### 1.4 In-situ laboratory SAXS: data analysis

The results of long-spacing, *L,* coherence length, *H,* and lamellar crystal sizes, *D,* as a function of applied tension and temperature, are summarized in Table 5 for the PCL crystals and in Table 6 for the P3HB α-crystals.

**Table 5:** Structural parameters extracted from peak (1), which are attributed to PCL crystals.

Stress (MPa)	T (°C)	peak (1) position (°)	long spacing *L* (nm)	peak (1) mer. width (°)	coherence length *H* (nm)	peak (1) trans. width (°)	lamellar diameter *D* (nm)
0	25	0.63	14.1	0.32	25.0	0.26	30.4
32	25	0.57	15.3	0.38	21.0	0.27	29.6
32	45	0.58	15.3	0.40	19.7	0.34	23.4

**Table 6:** Structural parameters extracted from peak (2), which are attributed to P3HB crystals.

Stress (MPa)	T (°C)	peak (2) position (°)	long spacing *L* (nm)	peak (2) mer. width (°)	coherence length *H* (nm)	peak (2) trans. width (°)	Lamellar diameter *D* (nm)
0	25	1.13	7.8	0.46	17.4	0.57	13.9
0	45	1.10	8.0	0.63	12.6	0.61	13.1
0	61	1.12	7.9	0.65	12.2	0.60	13.2
0	90	1.13	7.8	0.54	14.7	0.60	13.2
0	115	1.06	8.3	0.48	16.5	0.49	16.4
0	70	1.07	8.3	0.47	16.8	0.51	15.7
0	25	1.06	8.3	0.46	17.1	0.52	15.4
32	25	1.07	8.3	0.45	17.5	0.57	13.9
32	45	1.05	8.4	0.43	18.6	0.61	13.1
32	61	1.02	8.6	0.53	14.8	0.66	12.0
32	90	1.02	8.7	0.47	16.7	0.59	13.1
32	115	0.91	9.7	0.37	21.3	0.56	14.3
32	70	0.90	9.8	0.36	22.0	0.60	13.2
32	25	0.89	9.9	0.37	21.3	0.58	13.7

### 1.5 In-situ laboratory SAXS: meridional profiles

**Fig. 3:**
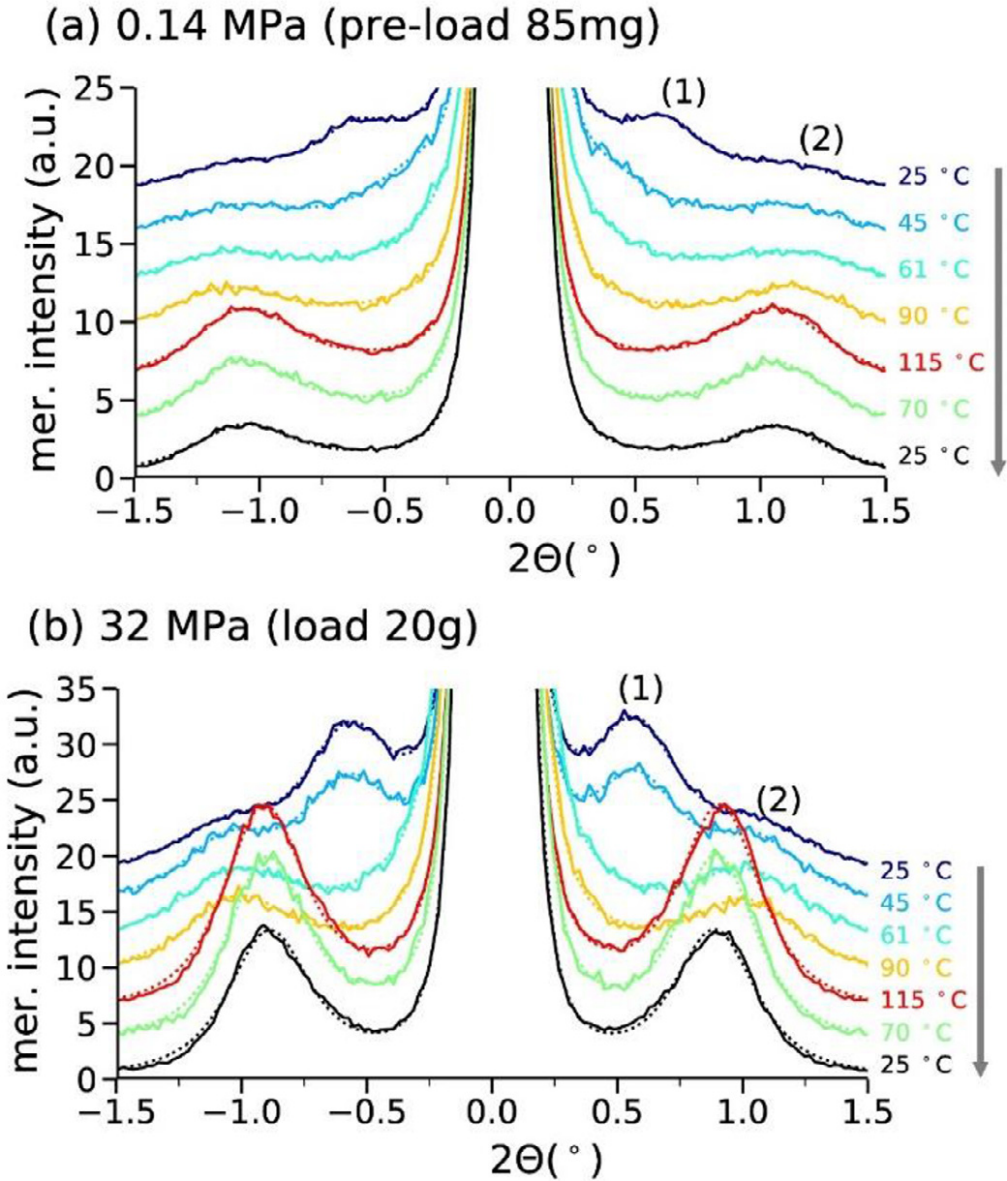
Meridional profiles for 0.14 MPa (a) and 32 MPa (b) at different temperatures. The arrow indicates the temperature sequence. Fits: dotted curves; measured data: solid curves.

All meridional intensity distributions of the SAXS patterns were individually fit with six Pearson VII functions (2 for the direct beam, and 4 symmetric peaks). The fits for meridional curves are shown as dotted curves, whereas the measured data is shown as full lines in Fig. 3.

### 1.6 In-situ laboratory SAXS: transversal profiles

**Fig. 4:**
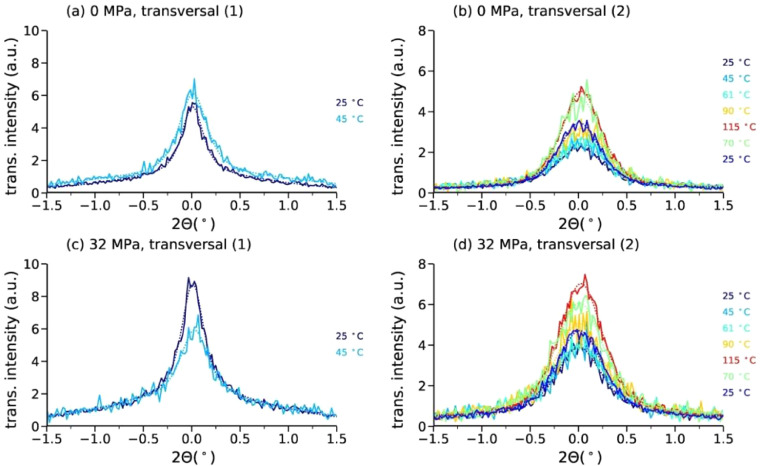
Transversal profiles across peaks (1) and (2) for 0 MPa (a, b) and 32 MPa (c, d). Solid lines correspond to measured data and the fits are shown as dotted curves.

Lamellar diameters, *D*, are extracted from the SAXS patterns by fitting the averaged intensity of the transversal areas to a Pearson VII function. The transversal intensity distributions are shown in Fig. 4 for the first and the second peak. The background in the transversal scans was fit with a broad Pearson VII function for peak (1) and with a linear function for peak (2).

### 1.7 WAXD/SAXS synchrotron data

**Fig. 5:**
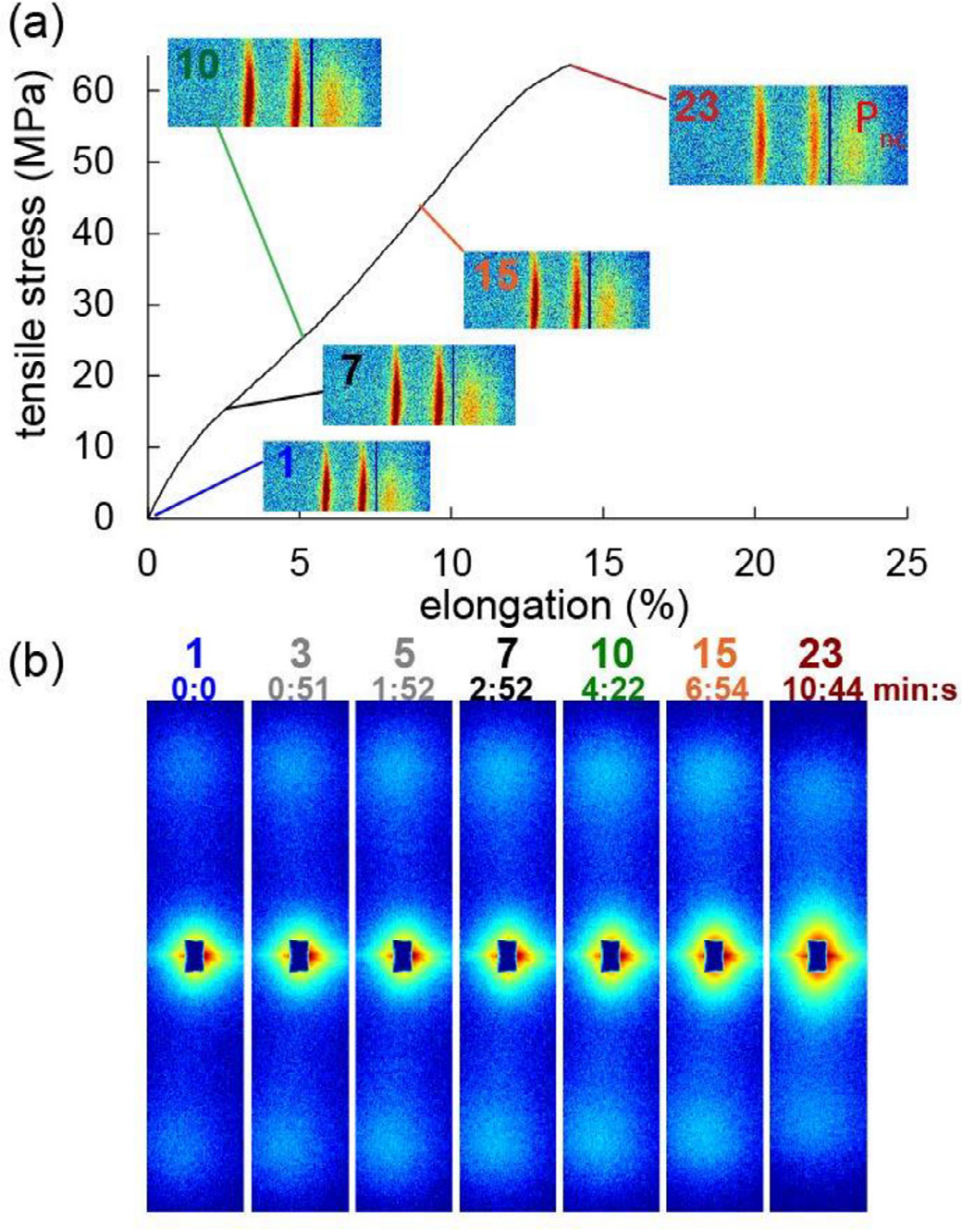
(a) Force-elongation curve for the high-stress fiber pre-annealed at 100 °C with 40 MPa. Insets: 2D-WAXD detector images (fiber axis is vertical). (b) Corresponding symmetrized SAXS profiles (fiber axis is vertical). The numbers indicate the image number.

**Fig. 6:**
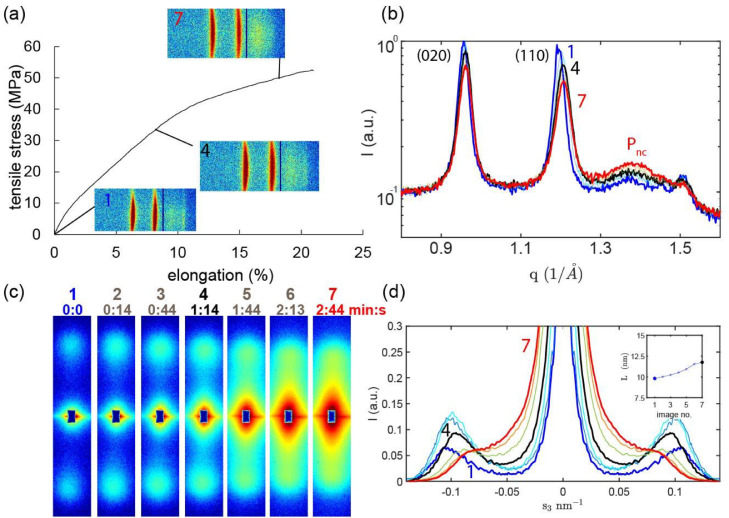
(a) Force-elongation curve for fiber pre-annealed at 130°C with 16 MPa. Insets: 2D-WAXD detector images (fiber axis is vertical). (b) Radially integrated equatorial scans. (c) Corresponding SAXS profiles (fiber axis is vertical). The numbers indicate the image number. (d) Meridional profiles. Inset: Long-spacing vs. image number.

**Fig. 7:**
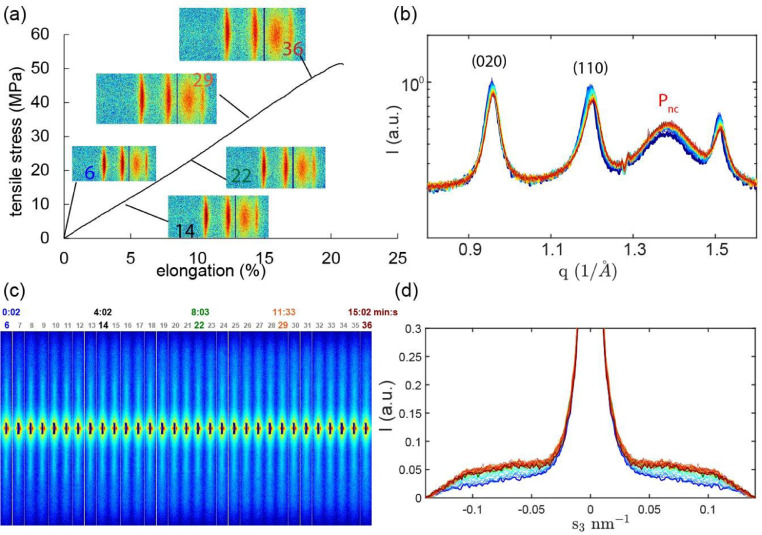
(a) Force-elongation curve of as-spun aged fiber. Insets: 2D-WAXD detector images (fiber axis is vertical). (b) Radially integrated equatorial scans. (c) Corresponding SAXS profiles (fiber axis is vertical). The numbers indicate the image number. (d) Meridional profiles.

The WAXD and SAXS data measured at the synchrotron during the post-drawing of the fiber pre-annealed at 100 °C with 40 MPa is shown in Fig. 5 and the data for the fiber pre-annealed at 130 °C with 16 MPa is shown in Fig. 6. The data of the aged fiber is shown in Fig. 7. The dark blue vertical line in the WAXD detector images is coming from the gap between two detector modules. Unfortunately, the fiber pre-annealed at 100 °C was tilted and thus the WAXD detector did not capture the full signal of the equatorial α-crystals and the P_nc_ peak. The P_nc_ phase is a highly oriented non-crystalline mesophase which consists of highly oriented tie molecules between α-crystals. However, the SAXS pattern was fully captured. All fibers have broken at smaller elongations than usual, most likely because of the high intensity of the x-ray beam. Furthermore, the aged fiber was measured with a higher frequency (every second) of the x-ray pulse.

## 2 Experimental Design, Materials, and Methods

### 2.1 Tensile tests of stress-annealed P3HB fibers

The materials and experimental methods for in-situ tensile tests on P3HB fibers have been previously described in detail in the article by Perret et al. [1]. Part of the text below has been taken from the said article and is therefore put into quotes.

"The fiber used for this study was melt-spun from modified P3HB (density 1.2 g/cm3) provided by Biomer (Krailling, Germany) on a customized pilot melt-spinning plant originally built by Fourné Polymertechnik (Alfter-Impekoven, Germany). A take-up godet was mounted at an unusually short distance (0.75 m) from the spinneret. This godet prevented the crystallization of the extrudate in a randomly oriented state. The fiber was melt-spun with a draw ratio of seven from ready-to-use modified P3HB pellets. The P3HB pellets contained a nucleating agent (boron nitride (BN)) and a plasticizer (tri-n-butyl citrate (TBC)) as well as various other processing aids including poly-ε-caprolactone (PCL). The fiber diameter is about 90 µm. (…) Annealing of the aged P3HB fiber (seven years) was conducted under various loads (1g, 10g, 20g, 25g) corresponding to 1.6, 16, 32, 40 MPa at different temperatures (60, 80, 100, 115, 130 °C) in a furnace with hot air circulation. The fibers with low loads (1.6, 16 MPa) were kept at all temperatures for 60 minutes. For the high loads (32, 40 MPa) the fibers were also kept for 60 minutes in the furnace for temperatures ≤ 100 °C. Above 100 °C the fibers could only be kept for 5 minutes at 105 °C in the furnace, because longer annealing times or higher temperatures caused breakage of the fibers. The length of each monofilament was measured at room temperature with a weight of 1g before and after annealing in order to determine the change in filament diameter and thus the linear mass density."

"We have performed tensile tests after aging and annealing. The load-strain behavior of the fibers was evaluated using the Textechno STATIMAT ME+ (Herbert Stein GmbH, Germany) tensile tester with a 10 N load cell, following ASTM D2256. The tensile tests of the monofilaments were performed with a typical test length of 100 mm and with a constant elongation rate of 100 mm/min."

Sometimes it is also of interest to look at the true stress-strain curves. The true stress is taking into account the changes of the fiber cross-sections during the tensile measurement. The true stress, σ_T_, is linked to the engineering stress, σ, and strain, ɛ, by the following equation [2]:

(1) $$\sigma_{T}=\sigma \left(1+\varepsilon \right)$$

The true strain is given by [2]:

(2) $$\varepsilon_{T}=\text{ln}\left(1+\varepsilon \right)$$

### 2.2 In-situ laboratory WAXD and SAXS measurements of P3HB fibers during stress-annealing

The experimental methods for in-situ WAXD and SAXS measurements on P3HB fibers during stress annealing have been previously described in detail in the article by Perret et al. [1]. Part of the text below has been taken from the said article and is therefore put into quotes.

"WAXD and SAXS patterns during in-situ annealing of three to four years aged fibers were recorded on a Bruker Nanostar U diffractometer (Bruker AXS, Germany) with Cu Kα radiation (λ = 1.5419 Å) and a VÅNTEC-2000 MikroGap area detection system. A beam defining pinhole of 300 µm was used. The WAXD and SAXS measurements were performed in two separate experiments with distances of 17.1 cm and 96.3 cm, respectively, between sample and active detector area. The heating stage H+300 (Bruker AXS, Germany) of the Nanostar diffractometer was used in order to study the effect of annealing. Single filaments were mounted on a custom-made fiber holder and different weights were attached at the end of the filaments in order to study the combined influence of heat and tension.

Two WAXD and SAXS experiments were performed with the in-situ heating stage: (A) The effect of low-stress annealing on the structure was studied by attaching a very small weight of 85 mg (0.14 MPa) to the end of the filament in order to keep the fiber straight during the measurement. (B) The influence of high-stress annealing on the structure was studied by attaching a weight of 20 g (32 MPa) to the filament. The stage with the filament was heated to various temperatures with 10 °C/min, and SAXS/WAXD patterns corresponding to the respective temperatures were recorded for typically 30 minutes or longer. During data collection, the temperature was kept constant by the heating stage control unit. (…)

The recorded WAXD/SAXS patterns were analyzed with the evaluation software DIFFRAC.EVA (version 4.2., Bruker AXS, Germany) and specially developed Python codes. (…) The intensities of all WAXD/SAXS patterns were corrected for exposure time and the thinning or thickening of the fibers from measured changes in the filament length."

#### 2.2.1 Analysis of WAXD profiles with azimuthal profiles: Orientation parameter

Peaks in azimuthal WAXD profiles were fit with Pearson VII distribution functions using Python codes [3] in order to extract the orientation parameter f(hk0) of the α-form crystals by applying the Herman's equation [4,5]. For f_(hk0)=_1, the (hk0) planes are perfectly aligned parallel to the fiber axis, and for f_(hk0)_=0, the crystals are randomly oriented.

#### 2.2.2 Analysis of SAXS patterns with meridional and transversal profiles

Long-spacings, coherence lengths and lamellar sizes were calculated by analyzing meridional and transversal areas of the SAXS pattern [4,6]. The lamellar long spacings, *L*, between α-crystals are extracted from the meridional peak position using the expression

(3) $$L=\frac{2\pi }{q_{\mathrm{LM}}}=\frac{\lambda }{\left(2\sin\theta_{\mathrm{LM}}\right)}$$

where $q_{LM}=\frac{4\pi }{\lambda }\sin\theta_{LM}$ is the scattering vector, *θ_LM_* is half the scattering angle at the lamellar reflection and λ is the wavelength.

The coherence length *H* along the fiber direction and lamellar stack diameters *D* are calculated from the width of the lamellar reflections along the meridian and from the width of the reflections in transversal profiles, respectively. To calculate these dimensions, the following Scherrer equation [7] is applied:

(4) $$size=\frac{0.9\lambda }{\Delta \left(2\theta \right)\cos\theta }\approx \frac{0.9\lambda F}{\sqrt{FWHM^{2}-b^{2}}}$$

Here, the *size* stands for either the coherence length *H* or the lamellar diameter *D, F* is the sample to detector distance, FWHM is the full width at half-maximum of the reflection, and *b* is the instrumental broadening (*b* ≈ 0). The equation makes use of small-angle approximations, cos *θ* ≈ 1.

### 2.3 In-situ synchrotron WAXD and SAXS of fibers during post-drawing after annealing

The experimental methods for in-situ synchrotron WAXD and SAXS measurements of pre-annealed P3HB fibers during tensile drawing have been previously described in detail in the article by Perret et al. [1]. Part of the text below has been taken from the said article and is therefore put into quotes.

"In-situ WAXD and SAXS measurements were performed at the cSAXS beamline at the Swiss Light Source of the Paul Scherrer Institute in Switzerland. The drawing at room temperature was performed using a TS 600 tensile stage (Anton Paar GmbH, Austria) with a 5 N load cell. A pre-annealed single filament was glued on top of supports and held by tensile stage grips. The single filament was elongated with an elongation rate of 0.5 mm/min until breakage while exposing the filament to the synchrotron x-ray beam every 30 seconds for 0.5 seconds. A vertically oriented Pilatus 300 K detector (Dectris LTD, Switzerland) was used to capture the equatorial region (0.55-2.75Å^-1^) of the WAXD patterns. Simultaneously, the SAXS patterns were measured with a Pilatus 2M detector [8]. A 2 m long flight tube was positioned in-between the SAXS detector and the drawing stage. The sample to detector distance was 2.139 m. The x-ray energy was 11.2 keV and the vertical beam spot size was about 25 µm."
